# Development of human dendritic cells and their role in HIV infection: antiviral immunity versus HIV transmission

**DOI:** 10.3389/fmicb.2013.00178

**Published:** 2013-07-09

**Authors:** Yasuko Tsunetsugu-Yokota, Mahmod Muhsen

**Affiliations:** Department of Immunology, National Institute of Infectious DiseasesTokyo, Japan

**Keywords:** DC–T transmission, HIV, DC subsets, accessory proteins

## Abstract

Although dendritic cells (DCs) represent a small cell population in the body, they have been recognized as professional antigen presenting cells and key players of both innate and acquired immunity. The recent expansion of basic knowledge concerning differentiation and function of various DC subsets will greatly help to understand the nature of protective immunity required in designing acquired immunodeficiency syndrome (AIDS) vaccines. However, human immunodeficiency virus (HIV) not only targets CD4^+^ T cells but also myeloid cells, including macrophages and DC. When HIV infects DC, its replication is highly restricted in DC. Nevertheless, even a low level of HIV production is sufficient to enhance HIV replication in activated CD4^+^ T cells, through antigen presentation activity by HIV-infected DC. Considering how antiviral immunity is initiated and memory response is maintained, such efficient DC–T cell transmission of HIV should play an important role in the disturbed immune responses associated with HIV infection. Recently, accessory proteins encoded by HIV have been shown to interact with various proteins in DC, and thereby affect DC–T cell transmission. In this review, we summarize the current understanding about DC biology, antiviral immune responses and DC restriction factors, all of which will be important issues for the development of an effective AIDS vaccine in the future.

## INTRODUCTION

Since the discovery of dendritic cells (DCs) in the 1970s (Steinman, 2007), DC have long been recognized as: (1) professional antigen presenting cells expressing high levels of major histocompatibility complex (MHC) class II and other costimulatory molecules, (2) localizing to various tissues/organs, and (3) migrating to lymphoid tissues after antigen acquisition, to either initiate immune responses or induce tolerance by interaction with T cells (see review by Banchereau and Steinman, 1998).

In the 1990s, we (Tsunetsugu-Yokota et al., 1995) and several others demonstrated that DC are easily susceptible to human immunodeficiency virus type 1 (HIV-1) infection and can therefore also efficiently transmit virus to antigen-specific CD4^+^ T cells, in spite of low levels of virus production. These earlier studies lead to further research into the role of DC in the pathogenesis of HIV infection, because DC–T cell interaction is considered to better reflect the physiological mode of cell-to-cell HIV-1 infection *in vivo* as compared to T-to-T or T-to-adherent cell interactions, which involve env-mediated membrane fusion (Sattentau, 2008). As our laboratory previously demonstrated, the close contact site between DC–T cells is consolidated by the presence of T cell receptor binding to MHC–antigen complex, followed by the interaction and mutual signaling of various costimulatory molecules (Tsunetsugu-Yokota et al., 1997).

A substantial number of reviews, focusing on HIV-1 transmission from DC to T cells (Rinaldo and Piazza, 2004; Wu and KewalRamani, 2006; Piguet and Steinman, 2007) have illustrated distinct features of cell-to-cell transmission. However, although the majority of studies focus on the close contact as a virological synapse (VS: a neuronal synapse like cell–cell contact structure for spreading virus infection), most of them do not consider the importance of antigen-dependent DC–T cell interaction, which is well-recognized as an Immunological synapse (IS: a synapse like cell–cell contact structure for activating immune response).

In this review, we will summarize the current understanding of various human DC subsets based on several outstanding recent findings in DC biology and of antiviral immune responses initiated by DCs, and discuss about newly identified DC restriction factors counteracting HIV-1 accessory proteins (e.g., Vif, Vpu, and Nef), which may have an impact on (1) the susceptibility of DC to HIV-1 infection and (2) the transmissibility of HIV from DC to T cells via VS or IS.

## BIOLOGY OF DENDRITIC CELLS

DC originate from common myeloid precursor cells in the bone marrow, but are quite heterogeneous in terms of their localization, surface phenotype, and function. The major DC subsets are classical or conventional DC (cDC) and plasmacytoid DC (pDC). The development pathway and the lineage relationship of these DCs have been subjects of extensive investigation (see review by Steinman and Idoyaga, 2010). By analyzing bone marrow precursors *in vivo*, a current view of DC development and homeostasis has been established. In the bone marrow, monocytes and DC precursors (MDP) first develop into a common DC precursor (CDP) before continuing development into either a monocyte, or a cDC precursor (PreDC) and pDC, respectively (Fogg et al., 2006; Liu et al., 2009). PreDC, pDC, and monocytes subsequently migrate through blood to the spleen and lymph nodes, whereby preDC differentiate into cDC in the presence of Flt3L, and monocytes differentiate into either macrophages or DC, presumably depending on the inflammatory stimuli present (Cheong et al., 2010).

With regards to lineage, there is considerable difference between mouse and human DC subsets, as defined by surface markers. Five major DC subsets are known in mice: CD8^+^DC, CD4^+^DC, and pDC in lymphoid tissues, and CD103^+^ and CD11b^+^DC in non-lymphoid tissues (Steinman and Idoyaga, 2010). Alternately, the source of DC in humans is more limited, originating from either skin or blood. In human skin, three DC subsets have been identified: epidermal CD207^+^ Langerhans cells (LCs), CD14^+^ dermal DC, and CD14^-^CD207^-^CD1a^+^ DC. Amongst them, LC are the most potent inducer of Th2 cytokines and able to cross prime CD8^+^ effector cells (Klechevsky et al., 2008). With respect to the origin of LC localized in the skin epidermis, Sere et al. (2012) have recently reported that LC are replenished in two waves, one by monocyte-derived, short lived LC upon inflammation, and the other by non-blood monocyte origin with long life expectancy (Sere et al., 2012). In other tissues and blood, two subsets of human cDC are known, which express either CD1c (BDCA-1) or CD141 (BDCA-3). The other group, pDC, expresses human-specific pDC markers, BDCA-2 and BDCA-4, in addition to CD123 [interleukin-3 receptor (IL-3R)], and produces a large amount of type I interferon (IFN; Steinman and Idoyaga, 2010).

A unique and important function of DC is their cross-presentation ability by phagocytosing dead cells, tumor cells, or infected cells, which results in the activation of MHC class I-restricted effector CD8^+^ T cells (Bevan, 2006). A particular subset of DC is known to efficiently cross-present antigens. In mice, splenic CD8α DC are the most potent DC subset for cross-presentation, and the human counterpart of mouse CD8α has been recently identified (Bachem et al., 2010; Jongbloed et al., 2010; Poulin et al., 2010).

The minor DC population in human blood, CD141^+^ DC, shares several phenotypic and functional properties with mouse CD8α DC, such as: (1) the expression of DC natural killer (NK) lectin group receptor-1 (DNGR-1) or CLEC9A, a sensor of necrotic cells (Sancho et al., 2009), and (2) the selective expression of the chemokine receptor XCR1 (Bachem et al., 2010), transcriptional factor ATF (activating transcription factor)-like-3, and IFN regulatory factor 8 (IRF-8). CD141^+^ DC also express a high level of Toll-like receptor 3 (TLR3) and upon TLR3 stimulation, produce IL-12 and IFN-β. More recently, by using comparative genomics to align human and mouse cell types, Haniffa et al. (2012) demonstrated that cutaneous CD141^hi^ CLEC9A^hi^XCR1^+^ DC, which are closely related to blood CD141^hi^ CLEC9A^hi^XCR1^+^ DC, are much more potent at cross-presentation than LC. Taken together, in humans, CD141^+^DC, CD1c^+^DC, and pDC localize within lymphoid tissues, while the former two types of DC and CD14^+^ DC are present in non-lymphoid tissues. In addition, human tissue CD141^+^ DC are functional homologues of mouse CD103^+^(α chain of the α_E_β_7_ integrin) non-lymphoid DC, with high cross-presentation activity (Haniffa et al., 2012).

However, the relationship between DC and monocytes/macrophages can sometimes be confusing when based only on cell surface markers and functional properties. Two types of human monocytes are present in the blood: “patrolling” CD14^dim^CD16^+^ and inflammatory CD14^+^ CD16^-^ or CD14^+^CD16^+^monocytes, which resemble mouse Gr^-^ (Ly6C^low^) and Gr^+^ (Ly6C^high^) cells, respectively (Auffray et al., 2009; Cros et al., 2010). When tissue inflammation occurs, CD14^+^inflammatory monocytes migrate to the site of inflammation, and may differentiate into CD11b^+^CD14^+^ monocyte-derived DCs (MDDCs) as demonstrated in mice (Cheong et al., 2010).

Currently, four major DC subsets comprise and make up the category of DC: cDC, pDC, LCs, and MDDCs (Satpathy et al., 2012). A recent large scale collaborative study focusing on the transcriptional network of DC and monocyte/macrophage subsets, highlighted on some lineage-specific key transcriptional factors and the mutual relationship between DC and monocytes/macrophages (Miller et al., 2012), though the definition of DC by molecular signatures has raised further discussion (Hume et al., 2013).

**FIGURE 1:**
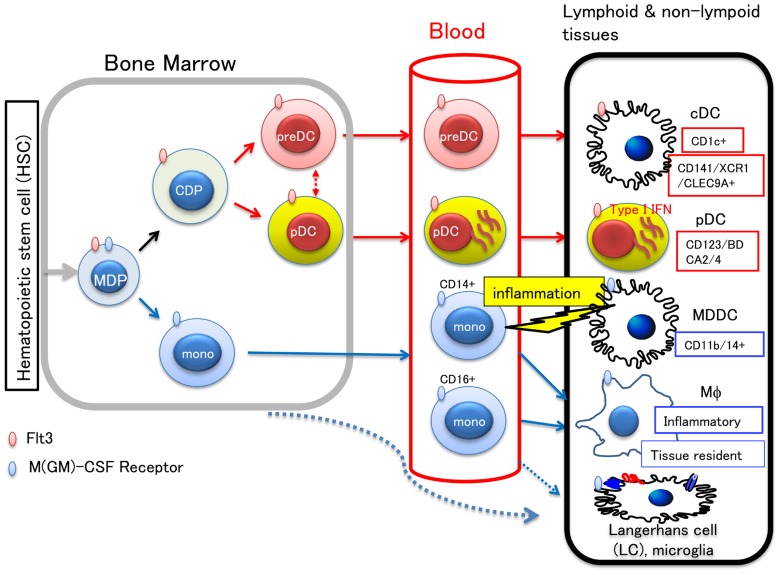
**Development of myeloid cells and lineage relationship between DC subsets.** Hematopoietic stem cells in the bone marrow differentiate into monocyte/macrophage and DC precursor (MDP), which commits to a distinct lineage cell, either becoming a monocyte or common DC precursor (CDP). CDP differentiates to preDC or pDC, and migrates to the peripheral tissues through blood. In the lymphoid or non-lymphoid tissues, preDC can become either of two types of cDC: CD1c^+^ DC and CD141^+^/XCR1^+^/CLEC9A^+^ DC, the latter of which has high cross-presenting activity. In the blood, CD14^+^ or CD16^+^ monocytes are also circulating. Blood monocytes are source of macrophages (Mϕ), LC, and microglias. The other LC precursor in the bone marrow, was also shown to migrate to the skin. Upon tissue inflammation, CD14^+^ monocytes migrate to the inflamed tissue and differentiate into either migratory MDDC or inflammatory Mϕ. It is known that the cells committed to become DC express Flt3, whereas those that commit to monocytes/macrophages express M-CSF receptor. Therefore, Flt3L and M-CSF are key molecules for differentiation and proliferation of DC and monocytes/macrophages, respectively.

Incorporating recent findings, the development and lineage relationship of human myeloid precursors is illustrated in **Figure 1**.

## OVERVIEW OF ANTIVIRAL IMMUNE RESPONSES IN ACUTE AND CHRONIC HIV INFECTION

The natural infection of HIV-1 mostly occurs through vaginal or rectal routes. Because these submucosal spaces are rich in DC, they have been argued to be the primary targets of HIV-1 infection (see reviews by Wu and KewalRamani, 2006; Piguet and Steinman, 2007). However, detection of HIV-1-producing DC in these tissues is rare, which is in contrast to the rapid and massive HIV/simian immunodeficiency virus (SIV) infection detected in CD4^+^CCR5^+^ memory T cells (Brenchley et al., 2004; Mehandru et al., 2004; Li et al., 2005; Mattapallil et al., 2005). Nonetheless, the scenario that mucosally residing, non-producing HIV-infected DC or phagocytosed HIV-1-infected DC migrate to regional lymph nodes, and subsequently spread HIV-1 infection there to contacting T cells, in association with nominal antigen presentation, well reflects what maybe occurring in lymphoid organs (Wu and KewalRamani, 2006; Piguet and Steinman, 2007).

In a chronic stage of HIV-1 infection, a major reservoir of latent HIV-1 infection is considered to be the circulating resting memory T cells, carrying integrated HIV-1 DNA (review by Pierson et al., 2000). When resting memory cells enter a lymphoid organ, they interact with resident DC, which may induce T cell activation and concomitant transmission of reactivated HIV-1 to DC. There is a possibility that newly HIV-infected DC can transmit virus to intact naïve or memory T cells. Although these HIV-1-infected T cells and DC can be eliminated by a cytotoxic T cell (CTL) response, a continuous cycle of such aforementioned events may cause intermittent surges of plasma viral load under chronic infection. Henceforth, lymphoid organs are considered to be major sites of HIV-1 production.

During the induction of primary immune responses in lymphoid organs, an inflammatory response can occur, as an innate immune mechanism at the invasion site, attracting a variety of immune cells, including T cells and monocytes. As previously described, so-called inflammatory monocytes are recruited into peripheral tissues subsequently, differentiating into inflammatory MDDC which can regulate immune responses locally (Cheong et al., 2010).

It is well-known that effector CD8^+^ T cells play an essential role regarding protection against HIV/SIV infection (Appay et al., 2008). Once infection primes in lymphoid organs, memory CD8^+^ T cells can act as effector cells by circulating in the blood, in order to migrate to infected tissues upon re-exposure, and quickly eliminate infected cells. The importance of long-term resident memory CD8^+^T (T_RM_) cells has been highlighted with respect to efficacy of the local memory response (Gebhardt and Mackay, 2012). By utilizing an elegant mouse model of herpes simplex virus (HSV) infection, memory CD8^+^ T cells were shown to be initiated in extra-lymphoid tissues, independent of migratory memory CD8^+^ T cells (Wakim et al., 2008). In this model, HSV-infected dorsal root ganglia (DRG) was surgically transplanted to naïve mice, because DRG is known to be latently infected with HSV, after acute virus resolution, and to be reactivated by surgical extraction. Stimulation of these CD8^+^T_RM_ cells was dependent on recruited DC in the DRG, and CD4^+^ T cell help was required. These CD8^+^ T_RM_ cells represent a self-renewing and highly protective population of memory T cells distinct from circulating memory CD8^+^ T cells, and express CD103 which is known to be widely expressed in non-lymphoid tissues (Gebhardt et al., 2009). Interestingly, a study using intravital two-photon microscopy revealed that while memory CD4^+^ T cells are trafficked rapidly, CD8^+^ T_RM_ cells are removing slowly in the original skin site during HSV infection (Gebhardt et al., 2011).

The functional properties of CD8^+^ T_RM_ cells have just begun to be elucidated in influenza virus infection. The innate antiviral function of IFN-induced transmembrane protein 3 (IFTM3) was first discovered by using RNA interference screening for factors modifying influenza virus infection (Brass et al., 2009). The IFITM family is: (1) made up of IFN-stimulated genes (ISGs) with diverse biological functions, (2) comprises of multiply closed members of four genes in both humans and mice, and (3) restricts various virus infections at a site of viral fusion (Diamond and Farzan, 2012). Recently, IFITM3 was reported to be expressed in lung CD8^+^ T_RM_ cells after influenza virus infection, endowing greater resistance to the secondary influenza virus infection (Wakim et al., 2013). Sustained expression of IFITM3 is intrinsically regulated in CD8^+^ T_RM_, but not CD4^+^ memory T cells, and enhances the survival of CD8^+^ T_RM_ cells only at sites of viral infection. Although the involvement of recruited DCs on the activation of CD8^+^ T_RM_ cells is demonstrated (Wakim et al., 2008), the mechanisms in which memory and effector CD8^+^ T cells develop and localize to target peripheral tissue such as intestinal mucosa and the involvement of intestinal DCs need to be addressed in future for the development of effective anti-HIV vaccines.

**FIGURE 2:**
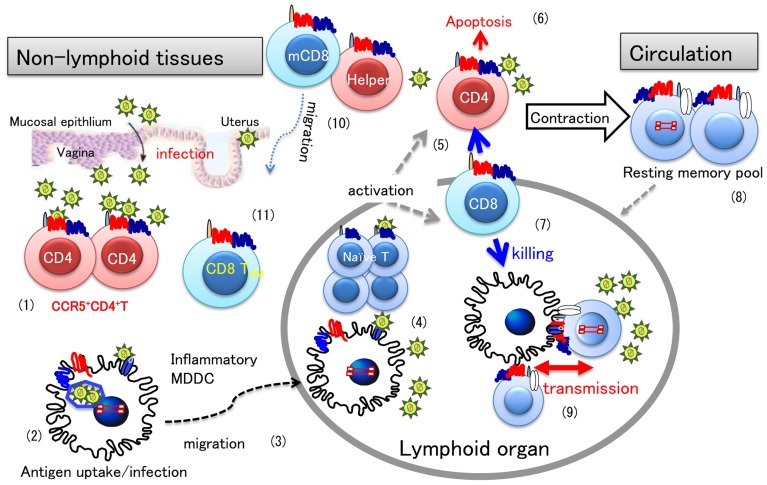
**HIV infection and T cell differentiation during primary and memory antiviral immune responses.** A general view of antiviral immune responses focusing on HIV-1 infection is illustrated. Once HIV invades the vaginal mucosa, infection mostly occurs in activated CD4^+^CCR5^+^ T cells (1). Resident DCs in the submucosa engulf virions or HIV-infected cells (2), and then migrate to the draining lymph nodes (3), where an antiviral immune response is initiated (4). In a primary immune response, antigen-specific T cells are differentiate into effector cells. During the effector phase, memory T cells are also produced, the mechanism of which is not fully understood. Once activated, CD4^+^ T cells are infected with HIV (5), however, most effector cells will die of apoptosis (6), or killed by effector CD8^+^ T cells (7). When virus-infected cells are eliminated in the infection site, the immune response enters into the contraction phase. After some time, memory T cells revert to quiescent or resting state (8) and circulate back to peripheral tissues for checking for next infection. Latent infection of HIV is known to occur in such quiescent memory CD4^+^ T cells. When these latently HIV-infected memory CD4^+^ T cells interact with uninfected DCs and T cells, for secondary antigen exposure, HIV-1 may spread to CD4^+^ T cells in the lymphoid organ through DC–T cell interaction (9). In the case of circulating memory CD8^+^ T cells (mCD8), they may quickly enter into tissues to eliminate the secondary infection (10). However, recent evidence indicate that tissue resident CD8^+^ T cells (T_RM_) (11) primed *in situ* play a more important role than circulating mCD8 in protective immunity, which may be a distinct feature from memory CD4^+^ T cells.

The overview of this section is illustrated as **Figure 2**

## DISTINCT SUSCEPTIBILITY OF DCs TO HIV: *CIS*- AND *TRANS*-INFECTION MODES

When MDDC are activated by various stimuli, such as lipopolysaccharide (LPS), TLR ligands, cytokines like type I IFN, DC express higher levels of MHC class II and other costimulatory molecules, thereby engaging in antigen presentation rather than antigen uptake. This maturation status and difference in lineage can profoundly affect the susceptibility of DC to HIV-1 infection (see review by Wu and KewalRamani, 2006).

Dendritic cell express various C-type lectin receptors (CLRs) which bind to HIV-1, such as DC-SIGN (dendritic cell-specific intercellular adhesion molecule-3-grabbing non-integrin), Langerin, and dectin (Turville et al., 2002), and recently discovered CLEC9A (Caminschi et al., 2008; Huysamen et al., 2008; Sancho et al., 2009). Each lectin receptor appears to have a unique and distinct function. HIV-1 transfer in *trans,* through the capture of virus by a CLR, such as DC-SIGN, occurs early in *in vitro* experiments (Cavrois et al., 2007; Dong et al., 2007; Izquierdo-Useros et al., 2007), and is designated *trans-*infection. However, DC lectin receptors are important molecules involved in the presentation of foreign antigens. In fact, the most of the HIV-1 virions captured by DCs were known to be rapidly degraded (Turville et al., 2004; Nobile et al., 2005). Moreover, the interaction of leukocyte-specific protein 1 (LSP-1), a protein directing internalized virus to the proteasome, with the cytoplasmic region of DC-SIGN may further facilitate the degradation of HIV-1 (Smith et al., 2007).

On the other hand, HIV-1 replication in DCs, but not DC-SIGN, is required for long-term transfer of HIV from DCs to CD4^+^ T cells (Lore et al., 2005; Nobile et al., 2005; Burleigh et al., 2006; Wang et al., 2007). Although DCs can support only minimal replication of HIV-1 (DC restriction, see Restrictions in DCs), it is considered that the antigen-dependent close DC–T cell contact, forming IS, would support the efficient virus transmission followed by massive virus replication in CD4^+^ T cells (Tsunetsugu-Yokota et al., 1995; Lore et al., 2005). We assume that such *cis*-infection is more likely to occur *in vivo*, as opposed to *trans*-infection.

**FIGURE 3:**
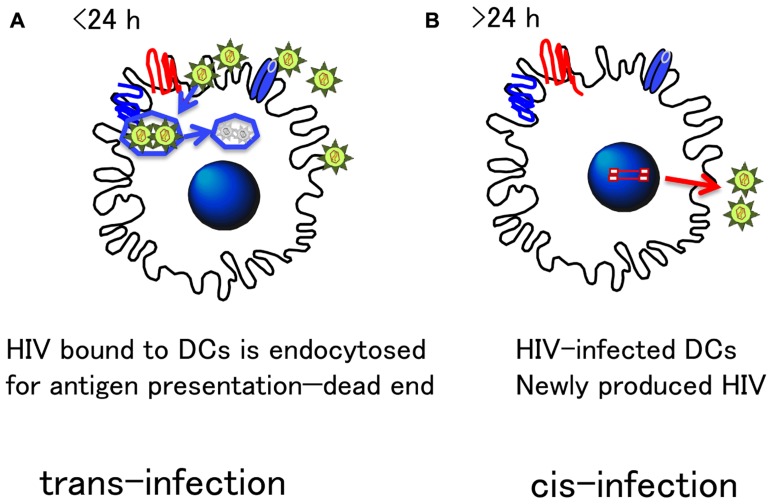
**Distinct HIV transmission mode by DCs: *Cis-* and *Trans-*infection.** There are two modes of transmission of HIV from DC to T cells. **(A)** *Trans-*infection: DCs express various receptors to bind foreign antigens, which is important to efficiently endocytose and process for antigen presentation. Such machinery is highlighted for HIV transfer *in trans* in *in vitro* experiments. However, because endocytosed antigens are quickly degraded within 24 h (Turville et al., 2004), infectious HIV particles may not retain their infectivity for a long time. Thus, the physiological relevancy of *trans*- infection *in vivo* remains unclear. **(B)** *Cis-*infection: On the other hand, because DC express CD4 and chemokine receptors essential for HIV infection, DC are naturally susceptible to HIV infection. Although HIV replication is limited in DC, their strong antigen-presenting activity allows HIV to replicate more in antigen-specific CD4^+^ T cells tightly interacting with DC. This DC–T cell interaction is a genuine immunological synapse (IS). The precise molecular mechanism of HIV transmission from DC to T cells has yet to be clarified, but the *cis-*infection mode, is what most likely occurs *in vivo*.

Thus, the *trans-*infection *in vitro* needs to be discriminated from HIV-1 transmission in *cis* or *cis*-transmission through genuine infection via DC as shown in **Figure 3.**

## THE ROLE OF TETHERIN IN HIV TRANSMISSION: IMMUNOLOGICAL SYNAPSE VERSUS VIROLOGICAL SYNAPSE

Various intrinsic antiviral mechanisms or factors have evolved in eukaryotes to fight against virus invasion (see review by Yan and Chen, 2012). To countermeasure these intrinsic cellular factors (restriction factors), HIV-1 evolved to encode several accessory proteins, such as Vpu, Vif, Vpr, and Vpx. BST-2/CD317/tetherin was recently identified as one such cellular factor, antagonized by HIV-1 Vpu (Neil et al., 2008; Van Damme et al., 2008). Normally, BST-2/tetherin inhibits virion release by anchoring virus at the cell membrane, but Vpu can directly bind to BST-2/tetherin, internalizing and degrading it, probably in the endolysosomal compartment (Arias et al., 2012).

It has been known for some time that vpu mutant virus can be efficiently spread by T cell to T cell transfer (Gummuluru et al., 2000) in a rapid turnover HIV-1 replication culture. As such, this observation raises the important question of whether Vpu affects cell-to-cell spread of HIV-1 by inhibiting the membrane tethering function of BST-2/tetherin? This issue was addressed under the setting of a VS, using HIV-infected cells directly contacting with an uninfected target cell system. Jolly et al. (2010) found that Vpu-defective HIV-1 disseminates more efficiently through cell-to-cell contact, despite the premise that BST-2/tetherin inhibits cell-free virion release, which corroborates with a previous report by Gummuluru et al. (2000), in which the authors speculated that BST-2/tetherin either mediates the accumulation of virions or regulation of VS integrity. On the other hand, Casartelli et al. (2010) studied virus transfer to T cells (targets) from HeLa or 293T cells, expressing a high level of BST-2/tetherin either naturally or by transfection (donors). They observed that BST-2/tetherin does not prevent VS formation, but assists the accumulation of Gag at the contact zone in the absence of Vpu (Casartelli et al., 2010). Important to note, however, is since the authors used mostly vesicular stomatitis virus (VSV)-pseudotyped HIV-1 for the infection of donors, their system is a transient infection event without new infectious virus production and reflect a cell-to-cell *trans-*infection as discussed in a previous section. Regardless, in both studies (Casartelli et al., 2010; Jolly et al., 2010), the accumulation of HIV-1 virions, in the presence of a high level of tetherin expression was observed.

Alternately, Kuhl et al. (2011) reported that a Vpu-mediated effect in viral spread among CD4^+^ T cells is independent of the extent of Vpu-mediated BST-2/tetherin cell surface downmodulation. They postulated the presence of an additional Vpu-independent mechanism for BST-2/tetherin cell surface downmodulation following HIV-1 infection in T cell lines, which is consistent with a report by Miyagi et al. (2009).

Vpu interacts with BST-2/tetherin to promote virion release, whereas BST-2/tetherin normally causes accumulation of virus particle on the membrane surface. However, the efficiency of cell-to-cell HIV-1 transfer or transmission appears largely dependent on experimental conditions. As previously described, DC–T cell contact is not equal to the VS form of contact. Therefore, we should perhaps consider the physiological function of BST-2/tetherin, under the setting of an antigen-mediated IS instead.

In this context, Coleman et al. (2011) reported that immature MDDC does not express BST-2/tetherin, however, after HIV-1 infection, BST-2/tetherin expression was upregulated by HIV-1 Nef. Therefore, HIV-1 transmission from DC to T cells does not appear to be restricted by BST-2/tetherin (Coleman et al., 2011). However, because their target is the HUT/CCR5 T cell line and culture conditions favor *trans* infection (immediate co-culture after HIV-1 infection), the question of whether cell surface expression of BST-2/tetherin assists or inhibits virus transmission to CD4^+^T cells via IS needs to be clarified.

## RESTRICTIONS IN DCs

**FIGURE 4:**
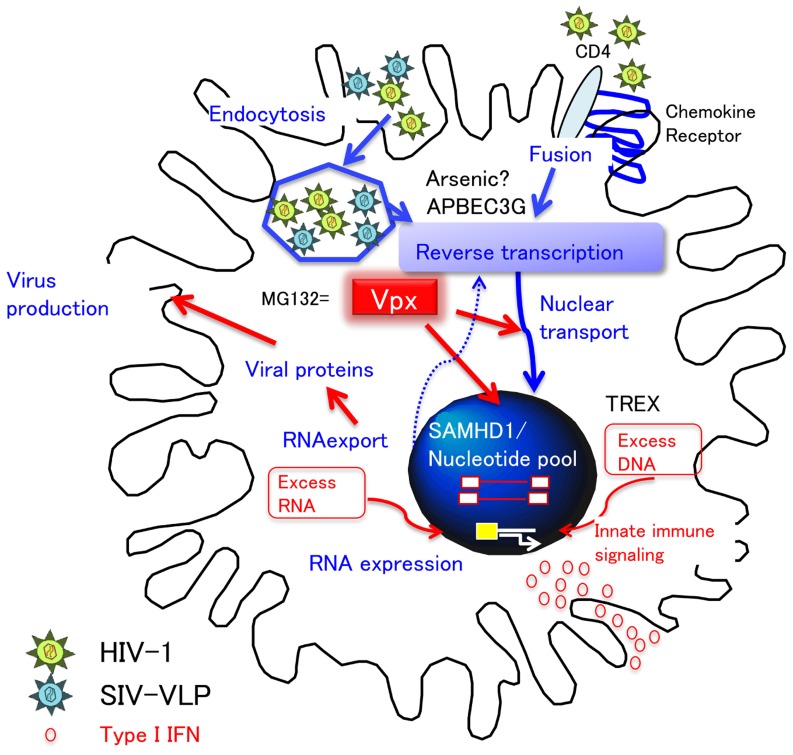
**DC restriction and type I IFN response.** HIV-1 utilizes CD4 and chemokine receptors for entry. The restriction of cDC to HIV-1 infection occurs at the entry (fusion) and post-entry level. Of note, in order to investigate the step at the post-entry level, VSV-pseudotyped virus is frequently utilized, which enters cells by endocytosis. After entry, APOBEC3G and arsenic sensitive factor inhibit reverse transcription. However, Vpx can counteract this restriction in DC by either degrading (1) SAMHD1-nucleotide pool-reverse transcription pathway or (2) nuclear transport. In addition, because the proteasome inhibitor MG132 works in a similar fashion to Vpx, it is possible that Vpx interferes with the proteasomal degradation step of endocytosed virions by degrading DC factors other than SAMHD1. Whatever the case, Vpx causes the accumulation of numerous virus products in the cytosol (red arrows). Importantly, the accumulation of viral DNA and RNA may trigger the innate immune signaling, resulting in type I IFN production, followed by the maturation of DC.

The poor replication of HIV in DC is partly explained by a block during virus fusion with MDDC (Cavrois et al., 2006). The restriction of HIV-1 infection in DC can also occur at a post-entry level. Several cell factors known to interfere with HIV-1 infection and/or replication step in DC are illustrated in **Figure 4**. APOBEC3 was originally discovered as a potent intrinsic antiviral factor interacting with HIV-1 Vif (Sheehy et al., 2002). APOBEC3G (A3G) is a member of the cytidine deaminase family, which edits C to U in a single stranded HIV DNA, causing G-to-A hyper mutation of the HIV-1 genome. HIV-1 Vif counteracts this deaminase function by inhibiting A3G incorporation into virions and promoting A3G degradation by ubiquitination (Sheehy et al., 2003).

Myeloid cells differentiate from monocytes to either macrophages or immature MDDC, and susceptibility to HIV-1 infection amongst these cell types has been inversely correlated to A3G expression, with highest A3G expression and lowest HIV-1 susceptibility in monocytes (Peng et al., 2007). In MDDC, A3G and APOBEC3F were shown to restrict HIV-1 infection at a post-entry step by Pion et al. (2006) and can be mainly ascribed to the deaminase-independent A3G function that inhibits reverse transcription (Bishop et al., 2008). However, Pion et al. (2006) also observed that arsenic trioxide enhanced the reverse transcription of HIV-1 in MDDC and CD1c^+^ blood cDC in an A3G-independent manner (Pion et al., 2007). Although the mechanism of arsenic in DC is currently unknown, these results suggest that cellular factors, other than APOBEC3, are definitely playing important roles in the restriction of HIV-1 replication, most likely at a reverse transcription step, in DC.

Coincidentally, a novel DC restriction factor was discovered from a study on Vpx. SIV virus like particles (VLPs) containing Vpx are known to enhance the expression of VSV-G-pseudotyped lentivirus in DC (Santini et al., 2000), and this finding lead to the identification of SAMHD1, a myeloid cell restriction factor (Caminschi et al., 2008; Hrecka et al., 2011). SAMHD1 is a potent triphosphohydrolase that converts deoxynucleoside triphosphates (dNTPs) to deoxynucleoside and triphosphate (Goldstone et al., 2011). Therefore, the current working hypothesis for the relationship between Vpx and SAMHD1 is that virus containing Vpx can replicate well in macrophages by degrading SAMHD1, which would otherwise reduce the dNTP pool and inhibit reverse transcription (Lahouassa et al., 2012). In SIV_SM_ or HIV-2 infection, Vpx was shown to be essential for viral replication and critical for reverse transcription of the viral RNA genome in macrophages (Fujita et al., 2008). However, a lack of Vpx in HIV-1 has negligible consequence with HIV-1, which can substantially replicate in macrophages. Therefore, Fujita et al. (2012) hypothesized that because of the lower activity of reverse transcriptase in HIV-2 versus HIV-1, HIV-2 had evolved to carry Vpx for compensation (Fujita et al., 2012).

However, it should be mentioned that immature MDDC do not support HIV-1 replication, in a similar manner, as macrophages do in R5-type HIV-1 infection (Tsunetsugu-Yokota et al., 1995) and that the susceptibility of HIV-2 containing native Vpx is even lower in MDDC and blood cDC (Smith et al., 2007). Furthermore, although remarkable Vpx-induced enhancement is observed in the case of VSV-pseudotyped lentivirus infection, it is not so obvious when native HIV was co-infected with SIV-VLP (Manel et al., 2010). Because it is known that the way virus enters via HIV envelope and/or VSV glycoprotein can lead to distinct outcomes in CD4^+^ T cells (Yu et al., 2009), the HIV envelope and its signaling in DC may contribute to different effects of Vpx.

There is no convincing explanation as to why the DC restriction, common to natural HIV-1 and HIV-2 infections, would necessarily be mediated by Vpx causing SAMHD1 degradation *per se*. Considering that the proteasome inhibitor, MG132, exerts similar effects with Vpx (Groot et al., 2006), we speculate that Vpx delivered into endosomes may inhibit endosomal degradation of endocytosed VSV-based lentivirus vector, which results in the release of numerous viral particles into the cytosol followed by production of a high copy number of RT products, thereby enhancing all subsequent steps of HIV-1 infection (integration, RNA synthesis, nuclear export, etc.). This scenario still needs to be proven and validated. Determination of whether Vpx induces the degradation of DC factors, other than SAMHD1, will prove to be an interesting future endeavor.

## Vpx AND TYPE I IFN

Another interesting feature of Vpx is that SIV-VLP containing Vpx induces a high level of Type I IFN in DC (Manel et al., 2010). Genetic diseases lacking SAMHD1 and TREX, a recently identified 3′-exonuclease which can suppress excess DNA accumulation in the cell (Yan et al., 2010), are known to develop similar autoimmune diseases due to a high level of type I IFN production (Lim and Emerman, 2011), indicating that these two distinct cellular proteins are important to regulate type I IFN responses. Thus, it is safe to assume that SAMHD1 degradation by Vpx can contribute to the induction of a high level of type I IFN production in DC. However, because Vpx enhances intracellular virus replication events as described in the previous section, excess accumulation of proviral DNA, as well as viral RNA, will occur, which might act as triggers for innate immune signaling in DC as depicted in **Figure 4**.

In contrast to other RNA viruses such as measles and influenza infection (Zilliox et al., 2006), HIV-1 infection, and other retroviruses also, do not induce type I IFN responses in DC (Luban, 2012). As regards to the mechanism, IRF-3, a pathogen-sensing pathway component, was shown to be depleted in HIV-infected T cells (Doehle et al., 2009) but not in DC, whereas Vpr-dependent inhibition of IRF-3 nuclear translocation was reported to occur in DC (Harman et al., 2011). The stimulation of DNA- and/or RNA-sensing pathway by Vpx may overcome this Vpr effect in DCs.

Finally, it is well-known that type I IFN is one of a multitude of cytokines which can induce the maturation of DC (Santini et al., 2000), and the maturation of MDDC has been shown to block HIV-1 infection at a post-entry level (Dong et al., 2007). However, in pDC, type I IFN production is induced by HIV-1 infection, as with other virus infections, resulting in inhibition on HIV-1 replication (Groot et al., 2006). Therefore, the lack of innate immune responses in cDC will be compensated *in vivo* in the early phase of HIV infection. Surprisingly, Schlafen 11 (SLFN11), a molecule induced by type I IFN, was shown to inhibit the translation of HIV-1 based on its virus-specific codon usage (Li et al., 2012). This finding indicates that there are still many undiscovered factors related to type I IFN signaling in DC which may be exploited to fight against retrovirus infections. Alternately, from the HIV side of things, by counteracting these restriction factors, HIV is able to establish a low level infection in DC. However, once type I IFN is induced in DC, it will help to generate more potent antigen-presenting DC, as demonstrated by Manel et al. (2010). Vaccine strategies utilizing Vpx for the enhancement of antigen presenting cell (APC) function in DC have already began and look promising (Durand et al., 2013).

## CONCLUSION

We now recognize that the majority of recently identified cellular factors interacting with HIV accessory proteins are related to the type I IFN or innate immune response. The remarkable antiviral activity of type I IFN has been well-known for a long time, but we are just beginning to understand its precise mechanism, which is not necessarily common to all cell types, especially in DC. It is has been postulated that HIV has evolved to escape from potent antigen presenting activity of DC, and took advantage of subverting within DC with a minimum level of replication, for easy transmission of virus to T cells, during the T cell activation process. As described in this review, our knowledge concerning the biology of various subsets of DC has advanced enormously. However, we need to further apply this basic knowledge and understanding to manipulate DC by placing them in crucial sites (tissues), within proper time frames, for the development ofa protective acquired immunodeficiency syndrome (AIDS) vaccine.

## Conflict of Interest Statement

The authors declare that the research was conducted in the absence of any commercial or financial relationships that could be construed as a potential conflict of interest.
